# Robust Non-Wetting PTFE Surfaces by Femtosecond Laser Machining

**DOI:** 10.3390/ijms150813681

**Published:** 2014-08-08

**Authors:** Fang Liang, Jorge Lehr, Lisa Danielczak, Richard Leask, Anne-Marie Kietzig

**Affiliations:** Department of Chemical Engineering, McGill University, 3610 University Street, Montreal, QC H3A 0C5, Canada; E-Mails: fang.liang@mail.mcgill.ca (F.L.); jorge.lehr@mail.mcgill.ca (J.L.); lisa.danielczak@mcgill.ca (L.D.); richard.leask@mcgill.ca (R.L.)

**Keywords:** femtosecond laser ablation, superhydrophobicity, PTFE, Cassie wetting, surface structure, biomimicry, insect wings

## Abstract

Nature shows many examples of surfaces with extraordinary wettability, which can often be associated with particular air-trapping surface patterns. Here, robust non-wetting surfaces have been created by femtosecond laser ablation of polytetrafluoroethylene (PTFE). The laser-created surface structure resembles a forest of entangled fibers, which support structural superhydrophobicity even when the surface chemistry is changed by gold coating. SEM analysis showed that the degree of entanglement of hairs and the depth of the forest pattern correlates positively with accumulated laser fluence and can thus be influenced by altering various laser process parameters. The resulting fibrous surfaces exhibit a tremendous decrease in wettability compared to smooth PTFE surfaces; droplets impacting the virgin or gold coated PTFE forest do not wet the surface but bounce off. Exploratory bioadhesion experiments showed that the surfaces are truly air-trapping and do not support cell adhesion. Therewith, the created surfaces successfully mimic biological surfaces such as insect wings with robust anti-wetting behavior and potential for antiadhesive applications. In addition, the fabrication can be carried out in one process step, and our results clearly show the insensitivity of the resulting non-wetting behavior to variations in the process parameters, both of which make it a strong candidate for industrial applications.

## 1. Introduction

As an old Chinese proverb goes, a lotus lives in the silt but not imbrued. Dirt particles are easily picked up by water droplets freely rolling over lotus leaves. This non-wetting behavior has found considerable research interest over the past decades. However, lotus leaves are only one of many natural surfaces that exhibit non-wetting and self-cleaning behavior. Nature has optimized plant and animal species for their respective living environments, such as the surfaces of plants [1,2], the wings [3,4,5] and legs of insects [6,7], or the skin of marine animals [8] and reptiles [9]. All of these exhibit prime examples for surfaces with specific functionalities, such as air-trapping, self-cleaning, anti-fouling and anti-bacterial characteristics. With an increasing world population and more and more pressing environmental problems researchers and policy makers are recognizing that learning from Nature as well as imitating and adapting the products of Nature’s century long design process can deliver solutions to everyday problems, which are often more sustainable, and rather preventive than reactive, in comparison to synthetic polluting disinfectants, cleaning agents and biocides.

A common characteristic of the above mentioned natural examples is their superhydrophobic wetting behavior, which is supported by physical surface features of different shapes and complexity depending on the organism and its environment. Microscale protrusions with nanoscale superimposed features [10] are as common as arrays of nanoscale rods, interconnected ridges or entangled fibers [3,4]. While different in shape, from a wetting perspective all these features can support air-trapping Cassie-Baxter wetting [11], where a water volume is suspended between surface features without completely penetrating the feature valleys due to an equilibrium of wetting (Laplace) and non-wetting (capillary) forces. Therewith, air is trapped between surface and liquid, and the actual contact area between liquid and surface is significantly reduced, which results in low adhesion. The robustness of the air-trapping wetting state is an important requirement for a surface to support and maintain advanced functionalities, such as self-cleaning and antifouling. More precisely under robustness we consider a surface’s ability to withstand transition from air-trapping superhydrophobicity to complete water penetration into surface valleys, which is characterized as Wenzel wetting, and results in considerably higher adhesion [12].

Biomimetic superhydrophobic surfaces that support air-trapping have received considerable attention in nanotechnology and biomedical applications [13]. Lu *et al.* (2006) have proposed a superhydrophobic fishbone microvalve design which supports air-trapping and therewith inhibits protein adhesion in the fabrication of a microfluidic biochip which was a concern when using traditional capillary valves [14]. Gentile *et al.* (2011, 2012) have shown that dilute solutions can be concentrated onto the liquid-supporting solid of air-trapping superhydrophobic surfaces to allow for detection of low concentration solutes which is of interest e.g., for the early detection of cancer cells [15,16]. Sousa and Mano (2013) have illustrated the fabrication of superhydrophobic paper and its application for various sustainable laboratory apparatus, which support the storage, transfer and mixing of aqueous media [17]. The above examples illustrate that there are many possible applications for extremely non-wetting surfaces. However, the fabrication of such surfaces is in many cases rather complicated, involving many process steps and demanding equipment that does not favor easy scale-up.

This project, inspired by natural superhydrophobic surfaces, had the particular goal to create a highly robust synthetic non-wetting surface through a relatively simple fabrication process for easy scaling-up and transfer to such industrial applications as outlined above. Ultra-short pulsed laser machining is a technology that fulfills the latter requirements, as it has already been shown to be successful in creating biomimetic surface features that support superhydrophobicity [18]. The dominating advantages of femtosecond (fs) laser ablation in comparison to other microfabrication methods are the non-contact optical machining process in combination with the short pulse duration which enable the machining of complicated 3D features while causing minimal thermal damage to the substrate material in a single process step [19,20]. Recent research efforts have particularly focused on the induced surface structures resulting from direct laser writing of metallic and semiconducting materials, while less research has been carried out on the structures resulting from femtosecond laser ablation on polymeric substrates [18,19,20,21].

A polymer of particular interest for biomimetic superhydrophobic surfaces is polytetrafluoroethylene (PTFE) due to its thermal stability, chemical inertness and low surface energy. The first report on laser ablation of PTFE was contributed by Küper *et al.* (1989) who presented that ablation with a fs UV excimer laser in air results in a roughness on the length scale of 1 µm on the ablated spot [22]. While Kumagai *et al.* (1994) showed high magnification electron microscope images of PTFE ablated with a Ti:Sapphire system in ultrahigh vacuum that show clean ablation edges [23], Adhi *et al.* (2003) report porous walls for holes drilled in air with a femtosecond UV excimer laser [24]. Lippert and Dickinson (2003) provide a comprehensive overview of surface features created by ablation on various polymers. The surface features observed on PTFE are described as fractal-like [21]. Another study by Hashida *et al.* (2009) on expanded PTFE (ePTFE) shows a microporous fiber network before and after irradiation with a Ti:Sa laser system [25]. Very little work is published that focuses on the wettability of PTFE surfaces after fs laser machining. A study by Wang *et al.* (2003) considers adhesion by contact angle measurements on PTFE channels ablated with a Ti:Sapphire laser [26]. Their results showed increased adhesion on the laser ablated surfaces. Furthermore, the authors observed so called microcone features at low pulse numbers, which give place to clean cut surfaces when more than 5 pulses are applied. In contrast, recent work by Huang and Ming (2010) highlights that multipulse craters created by femtosecond laser irradiation show microfeatures of entangled fibers which behave in a superhydrophobic manner with contact angles of above 150° [27].

In this work we present how to fabricate biomimetic, robust non-wetting structures on PTFE surfaces by fs laser micromachining. In particular, larger surface areas of such structures are desired instead of multipulse craters to investigate the suitability of this machining process for industrial applications.

## 2. Results and Discussion

### 2.1. Femtosecond Laser Micromachining

PTFE surfaces have been micromachined with the intention to create surface structures that robustly support air-trapping Cassie-wetting. Samples were raster scanned under the stationary laser beam at various positions along the beam path.

#### 2.1.1. Homogeneous Surface Patterns

**Figure 1:**
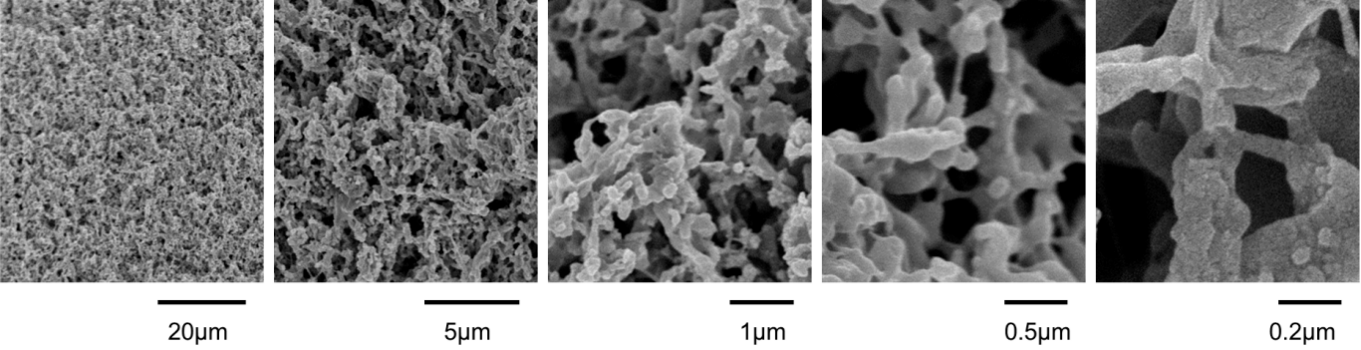
SEMs at different magnification of the surface pattern ablated by raster scanning with 100 mW and 89% overlap at a sample position of 2 mm in front of the focus.

Figure 1 displays an example of the characteristic entangled surface structures resulting from laser micromachining PTFE samples.

**Figure 2:**
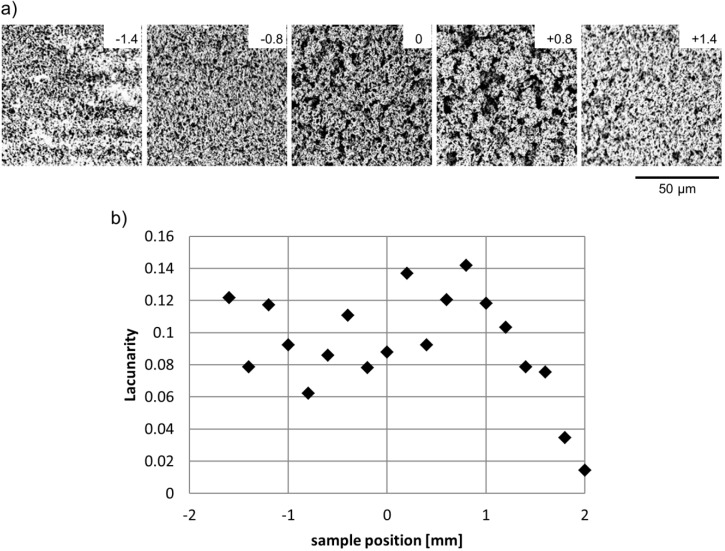
Structural characteristics of surfaces ablated by raster scanning with 180 mW and 93% overlap at different positions with respect to the focused beam waist (**a**) SEMs of the different surface patterns (numbers indicate distance from focus in mm); (**b**) Lacunarity.

The laser-ablated surface no longer exhibits a smooth polished surface, but rather a pattern which can be described as a forest of entangled thin fibers. This entanglement results in a porous surface layer composed of PTFE fibers and interconnected air channels. The fibrous structures show a certain similarity to expanded PTFE, which also shows a mesh-like microporous structure and has found wide application for waterproofing textiles (GoreTex™, Flagstaff, AZ, USA), as material for facial implants and filtration devices [28,29]. While ePTFE is made from stretching a PTFE film, the PTFE fibers are tightly connected to the solid bulk material. The degree of entanglement and the homogeneity of the ablated fibrous forest can be influenced through laser parameters. Figure 2 shows examples from an experiment, where samples were placed at different positions along the beam path while the same average laser power and raster scan line overlap was applied. In the following the focused beam waist position is denoted by 0. Sample positions given with positive numbers indicate the distance of the sample from the focused beam waist along the beam propagation axis towards the focusing lens, while sample positions behind the beam focus are labeled with negative numbers indicating the distance in millimeters to the focused beam waist.

The fiber entanglement and therewith the pore structure clearly varies with position, as also indicated by the surfaces’ lacunarity, as a measure of the surface’s fractal texture. At −1.4 mm behind focus the sample received barely enough energy to develop the hairy structure. The white spots are characteristic for the pristine polished PTFE surface, and hence signify surface areas that due to polishing imperfection were slightly lower than the remaining surface and therewith slightly further behind focus. When closer to the focus, the beam diameter decreases, which results in higher energy intensity, and thus in completely ablated surface patterns; the hairy features become more entangled the closer the sample is positioned to the focus. Interestingly, the degree of fiber entanglement is not symmetric around the beam focus as might have been expected based on optics theory. When the sample is placed slightly in front of the focus position (e.g., 0.8 mm) the fibers appear considerably more entangled compared to the position of same distance behind focus. Furthermore, lacunarity shows a maximum not at, but rather in front of, the focus position. These observations indicate that slightly in front of focus samples are subject to the highest effective intensity. With increasing positive distance to focus the entanglement, and thus the lacunarity, decreases again as the beam broadens, and while the threshold for onset of entangled structures was at −1.4 mm behind focus, it is at +2.9 mm in front of focus (additional SEM for the experimental series can be found in the Supplementary Information).

#### 2.1.2. Line Experiments

**Figure 3:**
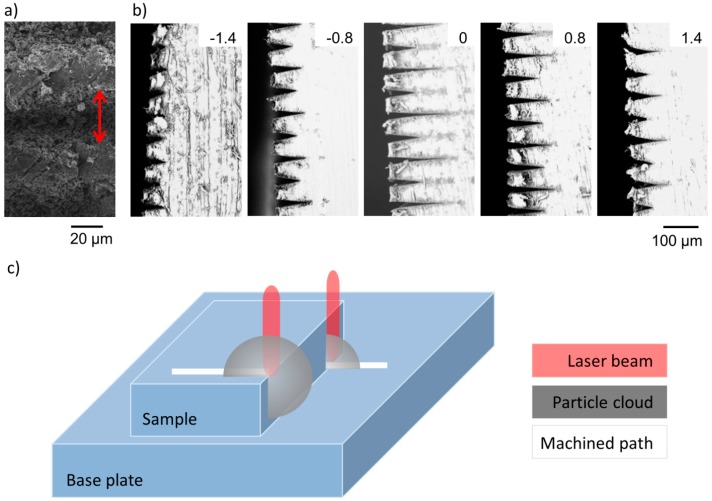
SEMs of lines ablated by raster scanning with 180 mW. (**a**) Top view machined at +2.4 mm (red arrow indicating line width); (**b**) Sample cross section at different positions with respect to the focused beam waist (numbers indicate distance from focus in mm); (**c**) Schematic of shielding when writing lines off (front) or onto the sample (back).

Line width experiments were carried out to further investigate the relationship of ablated surface structure and sample positions along the beam path. Figure 3 shows exemplarily the top view as well as cross sections of such raster scanned lines.

**Figure 4:**
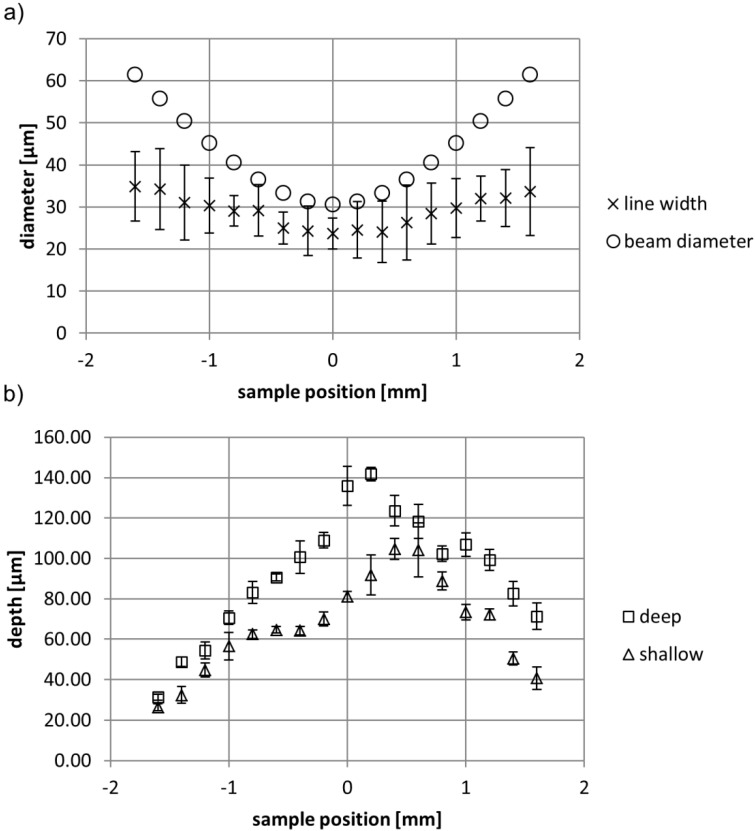
Line dimensions as a function of sample position for laser machining at 180 mW. (**a**) Beam width and ablated line width; (**b**) Line depth.

The top view image (Figure 3a) shows the characteristic fibrous structure on the ablated lines. Figure 3b illustrates that both line diameter as well as line depth vary with position along the beam path. When the sample is placed behind the beam focus (negative position) lines get increasingly deeper and narrower when approaching the focus (0). For sample positions in front of the focus (positive position) the inverse trend is observed. Figure 4 summarizes the measured width and depth results.

Figure 4a illustrates that the ablated line width is smaller than the calculated laser beam diameter. This result is consistent with literature and typical for the strong bonding and poor ablation characteristics of PTFE [23,24]. The ablated line width follows the symmetry of the beam diameter when the sample is moved away from the focus in either direction. Line depth SEMs (Figure 3) and data (Figure 4b), however, confirm the asymmetry observations from Figure 2.

Lines machined in front of the beam focus are deeper than their counterparts positioned at equivalent distance behind focus. The asymmetry in line depth (Figure 3b and Figure 4b) and in fiber entanglement (Figure 2) with respect to the focus position can be explained by particle plume shielding. When machining in front of focus (positive position) a laser pulse gets attenuated by the ablated particle cloud of the last pulse. However, for sample positions behind focus the laser beam energy is not only absorbed by the particle cloud of the last pulse but also by the ionized air at focus, which accordingly leads to a lower effective intensity reaching the sample surface positioned behind focus in comparison to the one positioned at equivalent distance in front of focus. Yet, this shielding effect is only noticeable from surface analysis by SEM and depth measurements and not from width measurements. We assume that this is an artefact of our measurement method as the irradiated line edges show the characteristic forest of entangled thin fibers (Figure 3a), which complicates exact width determination. This difficulty is also mirrored in the standard deviation to the measurements as shown in Figure 4.

Another interesting observation resulting from line experiments is that every second line is deeper than the line before (Figure 3b and Figure 4b). The difference in depth of deep and shallow lines is particularly large around focus, while far behind focus this difference almost levels out. This peculiarity can be explained when considering the raster scan method applied in these experiments. The sample was moved under the laser beam, so that the beam scanned across the edge of the sample. Therewith the beam, which ablated a sample surface e.g., at focus, scanned across the edge and ablated the PTFE back sheet mounted behind the sample of interest on the translation stage as illustrated in Figure 3c. The surface of this back sheet was placed ~1.14 mm (sample thickness + double sided tape) beyond the actual sample of interest. After a certain vertical displacement the next line was machined from the back sheet onto the sample surface of interest. Considering that each subsequent laser pulse will interact with the expanding particle plume of the last pulse [30], more laser energy gets absorbed by the particle cloud by scanning off the sample than when moving onto the sample (Figure 3c). When moving onto the sample the next pulse can be considered almost unaffected by the particle plume that originated from the last pulse on the back sheet. Thus the first pulses on the actual sample ablate the latter with a higher effective intensity, since particle screening from previous pulses is not relevant yet, which results in deeper observed line width at the cross section. Accordingly, the shallow line depth results are more representative when considering continuous raster scanning of a flat sample surface. The additional attenuation by the ionized air at focus for samples positioned at negative positions is more severe at already lower energy levels, as can clearly be seen from the shallow lines in Figure 4b. About 10 um shallower lines are observed for positions behind focus in comparison to positions of equivalent distance in front of focus. Also the greatest depth for shallow lines was achieved about 0.5 mm in front of the focus, which corresponds to the observations from Figure 2 of highest entanglement. At this region of highest ablation intensity (about 0.5 mm in front of focus) the degree of fiber entanglement and machining depth are relatively insensitive to varying sample position by ±0.2 mm, which indicates the robustness of the resulting surface structure to variations in laser parameters.

#### 2.1.3. Heterogeneous Surface Patterns

**Figure 5:**
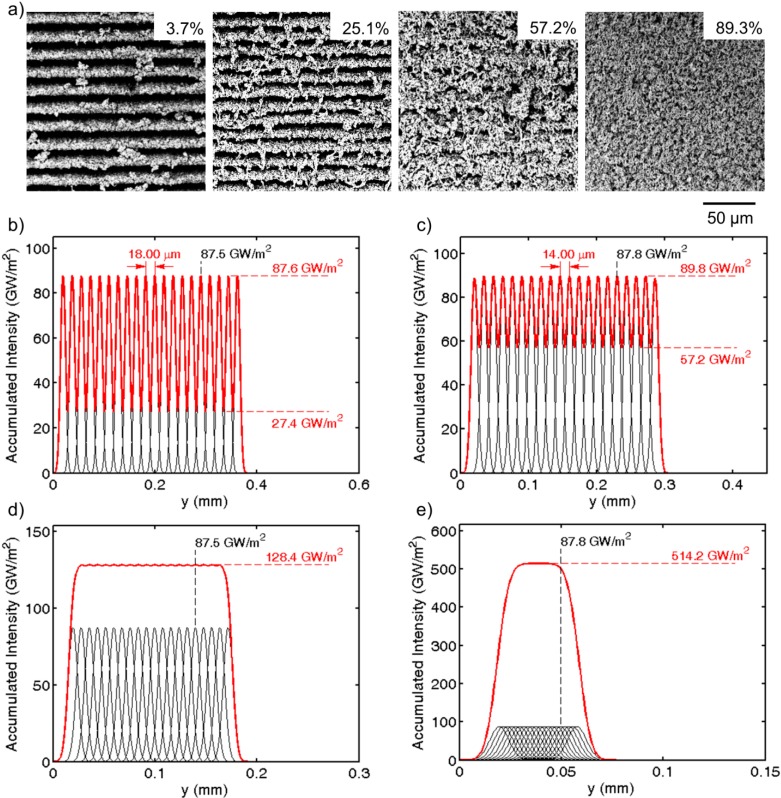
The effect of overlap on resulting surface patterns. (**a**) SEMs of surface patterns ablated by raster scanning with 100 mW (ablated line width of 18.69 μm) at focus with different settings of overlap; (**b**–**e**) Accumulated Intensity models for (**b**) 3.7%; (**c**) 25.1%; (**d**) 57.2% and (**e**) 89.3% overlap.

The data from the line width experiments was used to adjust line overlap for the raster scan of larger areas of the PTFE sample surfaces to better understand the influence of all machining settings. The resulting structures for varying overlap machined at focus are shown in Figure 5a.

At low overlap (3.7%) the surface shows distinct trenches (Figure 5a). The average spacing of these trenches was determined to be 18.16 μm. With increasing scan line overlap fibrous structures start to bridge the trenches, and with further increase of overlap the fibers get thinner, entangle and the trenches start to disappear (57.2%). At very high overlap the surface shows no trace of trenches anymore but is homogeneously covered by a forest of entangled thin fibers (89.3%), as shown in Figure 1 and Figure 2.

The observed trench distance can actually be predicted from the laser process parameters, as shown in Figure 5b–e. The accumulated intensity, to which the surface is subjected, can be modelled based on the applied raster scan machining process with a Gaussian laser beam. The black curves indicate the laser intensity applied to individual scanned lines, while the red curve sums these line intensities considering the scan line overlap (a detailed model description can be found in [31]). The modelled distance of the peak intensity from line to line corresponds to the measured values from experiments; Figure 5b predicts 18 μm, while the measured average was 18.16 μm. Similarly, Figure 5c models the accumulated intensity for 25.1% overlap, and the predicted distance of 14 μm corresponds reasonably well to the average measured trench distance of 14.47 μm. The model further illustrates that with increasing overlap the accumulated intensity profile becomes smoother. For an overlap of 57.2% the accumulated intensity (red curve in Figure 5d) barely shows a wavy profile. The result of this slight waviness is mirrored in the not quite homogeneous fibrous forest in Figure 5a. For an overlap value of 89.3% however, the forest appears homogeneous and the intensity profile (Figure 5e) shows a flat profile. With this understanding intensity modelling can be used as a tool to design desired non-homogeneous or homogeneous surfaces.

Overall, the above described femtosecond laser experiments on PTFE have clearly shown that the ablated surface shows a characteristic forest of nanoscale fibers. Whether ablating lines or larger areas the fiber entanglement is a function of laser intensity, which can be adjusted by positioning the sample with respect to focus or by adjusting average laser power. The structure homogeneity of area scans can be influenced by adjusting overlap. In particular the homogeneous surfaces are considered in more detail for non-wetting and bioadhesion applications in the following to assess the surfaces biomimetic potential.

### 2.2. Wetting

From a biomimetic standpoint the above presented homogeneous surface structures resemble the ones found on insect wings, such as e.g., those of the lace wing, mayfly or dragonfly [3,4,5]. Such insect wings exhibit a fibrous structure that has been described as “interconnected netting composed of ridges” [4], and they are known for their resistance to wetting with contact angles around 150° [3,4,5] as well as their antimicrobial properties [4]. Our laser-created structures, which we describe as forests of entangled thin fibers, exhibit fibers of similar dimensions as the natural examples introduced above. Figure 1 clearly shows that our PTFE fibers are of about 50–100 nm in width, whereas entanglement nodes reach diameters of about 0.5 µm. These dimensions correspond well to the typical feature dimensions observed for the dragonfly (65–350 nm) and the mayfly (65–950 nm) found on the wings of lace wing, mayfly and dragonfly [4]. The entanglement of the individual PTFE fibers of our surfaces shows great similarity to the fiber network observed on certain natural surfaces, such as the back of the ramee leaf and the surface of the Chinese watermelon [32]. While these natural plant surfaces exhibit a unitary smooth fiber surface, our surfaces rather show structural hierarchy with nano-roughness on the fiber surfaces (Figure 1) as seen for the structures on highly hydrophobic insect wings [5]. Considering the inherent hydrophobic nature of PTFE and the porous fibrous structures resulting from femtosecond irradiation, an air trapping wetting state is expected for our biomimetic surfaces. Before carrying out detailed wettings studies, it was confirmed by FTIR spectroscopy that the surface chemistry of PTFE did not change through the femtosecond laser micromachining, which is consistent with literature [24] (refer to supplementary material for spectra). Wetting experiments were carried out by drop impact and contact angle measurements. The surfaces were further sputter coated with gold to uncouple the respective contributions of surface structure and surface chemistry on the resulting wetting state and to test the wetting behavior for robustness.

#### 2.2.1. Drop Impact Experiments

**Figure 6:**
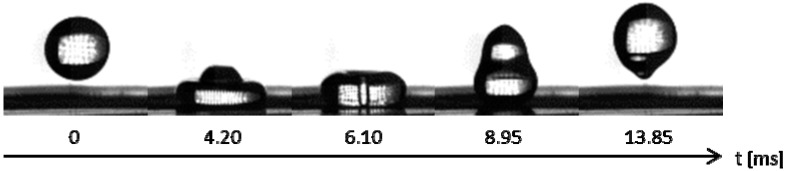
Drop impact sequence on PTFE laser-machined with 100 mW and 90% overlap at focus.

To test the wettability and robustness of the created structures, drop impact experiments with water were carried out on fibrous PTFE and subsequently gold coated surfaces. The behavior of both surfaces under drop impact was very similar: drops bounced off the surface and did not leave any water behind on the sample. Figure 6 illustrates the impact sequence on PTFE. Videos of experiments on the coated and uncoated laser-ablated surfaces are available in the Supplementary Material.

The experiments showed that the created surface structure behaves in a highly superhydrophobic manner. An impacting droplet is completely repelled and no water remains on the surface.

#### 2.2.2. Contact Angle Experiments

To test robustness and to discriminate between the goal-coated and uncoated laser-machined surfaces, contact angle experiments were performed with various liquids of different surface tension. The results for polished and laser-machined surfaces with and without gold coating are summarized in Table 1.

**Table 1:** Results of contact angle experiments; surfaces were laser machined with 100 mW and 89% overlap at focus.

Liquid	Surface Tension	Coating	Surface Structure	Sessile CA (°)
Water	72 mN/m	x	flat	107 ± 2
			fibrous	151 ± 7
		gold	flat	77 ± 4
			fibrous	148 ± 6
Glycerol	63 mN/m	x	flat	96 ± 8
			fibrous	135 ± 6
		gold	flat	74 ± 6
			fibrous	123 ± 7
Ethylene glycol	47 mN/m	x	flat	78 ± 4
			fibrous	133 ± 7
		gold	flat	60 ± 5
			fibrous	97 ± 7
Propylene glycol	36 mN/m	x	flat	71 ± 4
			fibrous	124 ± 3
		gold	flat	48 ± 4
			fibrous	31 ± 4

The high hydrophobicity of the fibrous structure with and without gold coating samples was confirmed. Uncoated laser-treated PTFE surfaces also showed highly non-wetting behavior (contact angle >120°) in contact with the other organic test liquids. Overall, fibrous surfaces with gold coating exhibit lower contact angles than uncoated fibrous surfaces for the different organic liquids, which was expected based on the material characteristics of gold and PTFE. Water, glycerol and ethylene glycol show consistently higher contact angles on the fibrous surfaces with and without coating in comparison to the respective flat surfaces. While ethylene glycol is wetting the flat PTFE and gold coated surfaces (contact angle <90°), the laser-created fibrous surface shows contact angles larger than 90°, which indicates the existence of air-trapping Cassie wetting. Propylene glycol, however, wets the gold coated fibrous surface more than the uncoated one; the fibrous surface structure reduced the average sessile contact angle from 48° to 31°. This is an indication for collapsed air pockets and complete surface wetting according to the Cassie theory. Thus, the threshold for wetting robustness for the gold coated laser-created surfaces lies at a liquid surface tension below 47 mN/m. Furthermore, the experiments showed that the high oleophobicity observed for the fibrous PTFE clearly results from a combination of the surface chemistry and surface structure.

### 2.3. Bioadhesion

**Figure 7:**
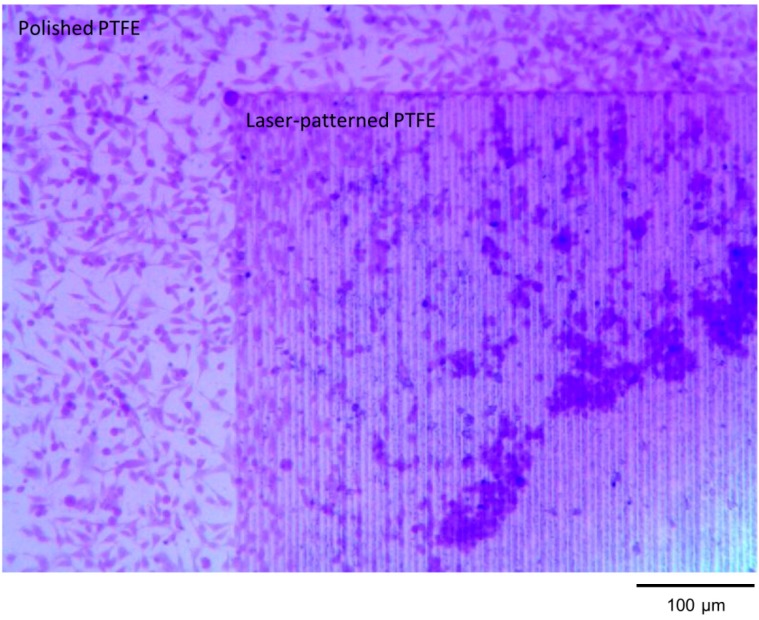
Microscope image of HeLa cell culture test on a laser-ablated PTFE surfaces with 100 mW and 60% overlap at focus.

Since our laser-created surfaces resemble anti-microbial insect wing surfaces in their homogeneous fibrous structure and their non-wetting character as outlined above, exploratory bioadhesion tests were performed to exemplarily test the response of biological organisms to the laser-ablated surfaces. The adhesion pattern of an immortalized human cervical cancer cell line (HeLa cells) clearly illustrates the air trapping and low adhesion potential of the laser-machined surfaces, as illustrated in Figure 7.

Healthy living cells can be identified by their characteristic elongated shape, while dead or unhealthy cells appear as round dots. Figure 7 clearly shows that more cells adhere to the polished PTFE as compared to the laser treated surface. The laser-patterned surface shows two distinct areas. The area that is bordering the polished surface supports the adhesion and growth of some cells. However, fewer and fewer living-healthy cells are found with increasing distance from the polished surface. The clear circular band of agglomerated cells separates a region that is occupied by very few cells (bottom right corner of Figure 7). This agglomeration of cells is likely cells growing on top of other cells that were able to anchor and adhere to the surface. Firmly attached cells try to migrate into open space but have difficulty anchoring and migrating within the treated area, creating a front of cells. The few cells, which can be seen in the interior circle are of round shape and appear to be unhealthy or dead. This interesting observation can be explained from a wetting perspective and by considering the machining process. As described in Section 2.2 the produced surfaces are clearly superhydrophobic and support air-trapping wetting behavior. The laser ablation results in the removal of material, so that the fibrous area is located below the polished PTFE surface. Furthermore, mainly dead or unhealthy cells are observed on the fibrous surface, which indicates that the high hydrophobicity of the surface repelled the cell culture medium during the growth and incubation of the cells. Similarly, the work of Alves *et al.* (2008) on rough casted PLLA films and the research of Ranella *et al.* (2010) on laser-structured titanium surfaces have shown that cells hardly adhere to biomimetic superhydrophobic surfaces which support air-trapping while cell adhesion is favored by hydrophilic non-structured substrates [33,34].

## 3. Experimental Section

Commercial PTFE sheets (1.1 mm thick, McMaster-Carr, Elmhurst, IL, USA) were polished by 600 and 1200 grit sandpaper to an average roughness of 500 nm before laser irradiation. A Ti:sapphire femtosecond laser with M^2^ of 1.2, 800 nm wavelength, 4 W maximum output power, 10 kHz repetition rate and 85 fs pulse duration was used in our experiments. The sample was mounted on a computer controlled translation stage which moved the sample under the Gaussian laser beam. A PTFE sheet, mounted between the stage base and the sample, ensured that samples did not get contaminated by nanoparticles from other material when machining across the sample edges. The beam power was reduced to the desired level by a half-waveplate and a beam splitter. A 100 mm focal lens was used to focus the 4 mm Gaussian beam to the desired spot size. Lines and patches, fabricated by raster-scanning at 4 mm/s, were ablated by varying power, scan line overlap and the sample’s position with respect to focus. The resulting surface structure was examined by scanning electron microscopy (SEM) (Phenom FEI; Hitachi SU-8230 CFE-SEM; Tokyo, Japan). Fractal analysis was carried out by differential box-counting and using a Matlab code developed by Al-Kadi and Watson and outlined in [35]. The fractal dimensions of our surfaces were found to be between 1.5 and 2, which is above the topological dimension *D* = 1 for a 1D object but below *D* = 2 for a 2D object. Strictly speaking, our surfaces do not qualify as 2D fractals [36]. However, the fractal analysis and in particular lacunarity values serve well to quantify homogeneity and density of our surface features. Ablated width and depth measurements were determined from line experiments, which were conducted across the sample edge as to characterize the cross section. Depth measurements were taken from at least 4 lines machined onto and off the sample, respectively, while line width and trench distance data represent the average of at least 8 measurements.

Surface chemistry of pristine and irradiated PTFE was analyzed by FTIR. A 10 nm thick gold layer was sputter coated on selected samples to render the surface chemistry while maintaining the same structures.

Drop impact experiments were carried out by dispensing a drop of 2 mm diameter from 5 cm height. A high speed camera (Photron SA5; San Diego, CA, USA) captured the impact event using an 18–108 mm Macro Zoom 7000 lens (Navitar; Rochester, CA, USA). Sessile contact angles of the laser-irradiated surfaces were assessed by goniometry using 2 uL droplets of water, glycerol, ethylene glycol and propylene glycol.

Cell culture tests were carried out with HeLa cells cultured in Dulbecco’s Modified Eagle Medium (DMEM) supplemented with 10% Fetal Bovine Serum (FBS) and 1% penicillin/streptomycin. The cells were seeded onto the PTFE surfaces in a 6 well polystyrene cell culture dish and allowed to adhere overnight. The following day the culture medium was removed and the samples were washed with 1× Phosphate-buffered saline (PBS) before being fixed in 1% paraformaldehyde for 20 min. Afterwards, they were washed in PBS again and stained with 4% crystal violet for 20 min before a final wash with PBS. The samples were mounted in Dabco mounting medium and were imaged immediately on a Leitz Diaplan upright microscope (Wild Leitz, Wetzlar, Germany).

## 4. Conclusions

We have shown that a PTFE surface after raster scanning under a 800 nm femtosecond laser beam shows a fibrous surface structure, which is reminiscent of the surface features found on certain insect wings and plant surfaces. The laser micromachining process proved suitable not only to produce homogeneous but also heterogeneous surface structures. By overlapping raster-scanned lines various surface patterns can be achieved ranging from separated trenches to wavy and finally homogeneous surface areas; the resulting pattern can be predicted based on laser intensity modelling. While the degree of fiber entanglement is generally a function of the applied laser intensity, the structures resulting at the position of highest ablation intensity are relatively robust to changes in laser settings, which makes laser micromachining a competitive fabrication method for industrial applications. Similar to the natural surfaces, the laser-created surfaces are extremely non-wetting to water and highly non-wetting to organic liquids, as was confirmed in drop impact and contact angle experiments. Gold coating of fibrous PTFE surfaces confirmed that the non-wetting characteristics are supported by the joint contributions of surface chemistry and surface structure. Exploratory bioadhesion experiments further illustrated that the biomimetic surfaces are truly air-trapping and therewith successfully inhibit cell adhesion. Future research is required to fully assess the susceptibility of such biomimetic surfaces to long time cell and biofilm exposure. Finally, as a non-contact optical machining method the one-step process can easily be scaled up and is therefore suitable to be applied to a vast range of geometries and could possibly serve as an alternative to expanded PTFE membranes.

## Supplementary Files

Supplementary File 1
